# Concealed Insanity, as Illustrated by Case of Mark Gray*Read before the Chicago Medical Society, December 18, 1882.

**Published:** 1883-09

**Authors:** D. R. Brower

**Affiliations:** Chicago, Ills., Professor of Nervous and Mental Diseases at the Women’s Medical College, and Lecturer on Practice of Medicine at Rush Medical College (Spring Course)


### Selections.

---

CONCEALED INSANITY.—AS ILLUSTRATED BY CASE OF MARK GRAY*. By D. R. BROWER, M.D., Chicago, Ills., Professor of Nervous and Mental Diseases at the Women's Medical College, and Lecturer on Practice of Medicine at Rush Medical College (Spring Course).

Insanity is no moral agent—the disordered nutrition of the brain upon which it depends, does not in any way improve the ethical tone of the unfortunate victim. If the patient was inclined to lie or steal or dissimulate before his insanity, he is none the less so inclined after. That insane patients should, therefore, deceive those around them, by concealing their insanity when occasion seems to require it, is not inconsistent with such insanity. That they do it, is within the experience of all who have had much personal contact with them. The motives which prompt the insane to action are not necessarily different from those which influence the sane. A desire to escape from the confinement of a hospital for the insane, or to avoid the ridicule of those around, or to maintain control of their affairs, is the usual incentive to this concealment. Those who are successful have delusions that are not necessarily manifest in their daily life and conduct.

Instances of concealed insanity are numerous. Ingels† reports a case in which a systematized delusional lunatic concealed his delusions so well, that he was about to be discharged when an accident which excited his emotional nature and caused him to give vent to his delusions. In a second case, a man who was guilty

---

* Read before the Chicago Medical Society, December 18, 1882.

† *Annales et Bulletin de la Société de Medicine de Gaud.* August, 1868.

of very *bizarre* actions, was twice discharged, and twice recommitted in one asylum. On the third admission Dr. Ingles was able to determine, but only after a long conversation, that the patient's actions were from the first based on systematized delusions. But for an accidental emotional explosion, these delusions would not have been elicited. In a third case, a systematized delusional lunatic had delusions of persecution, but for several years had so conducted his business as to lead every one to regard him as of perfect mental integrity. To his mother he communicated his delusive ideas, and she accepted them as true, but ascribed his persecution to sorcery.

Meyer* reports a case in which a man was able to conceal his insanity from his friends, and this insanity was only detected on the explosion of a wild business scheme based on it. Spitzka† cites a case, in which a systematized delusional lunatic was so well able to conceal his insanity, that he was appointed guardian over his insane sister. Blanche‡ reports several cases. in which patients concealed their insanity to avoid being douched and subjected to restraint by Leuret, who attempted to treat insanity by intimidation.

Munro§ had a curious experience of this kind. A patient brought action against him for false imprisonment, and underwent a severe cross-examination without revealing any delusion. It was suggested to the judge (Mansfield) to ask him what had become of the princess with whom he corresponded in cherry juice, and immediately a group of delusions became manifest. The patient indicted Dr Munro a second time, but could not be led to say a single word on the subject which had led to the failure of his first indictment. Blanchford|| says, that patients may deny their delusions for the purpose of regaining liberty. Forbes Winslow¶ states, that Lord Ellenborough expressed in the course of a judicial enquiry his opinion, that a patient had perfectly recovered. The patient was detected speaking in Latin in order to conceal his delusion. Bucknell and Tuke** cite a

---

* *Allgemeine Zeitschrift fuer Psychiatrie.* Baud xxiii.

† *American Journal of Neurology and Psychiatry.* August, 1882.

‡ *Del etat actuel des alienes traitè par Leuret.*

§ Cited by Bucknill and Tuke, Psychological Medicine, p. 477.

|| Insanity and its Treatment, page 361.

¶ Obscure Diseases of the Brain and Mind.

** Op cit.

case in which a patient was able to conceal his delusion in conversation, but revealed it in his correspondence. Hammond* states, that the insane may conceal their delusions for a purpose.

†Haslam states concerning the insane that "they have sometimes such a high degree of control over their minds, that when they have any particular purpose to carry they will affect to renounce their opinions which shall have been judged inconsistent, nd it is well known they have often dissembled their resentment auntil a favorable opportunity has occurred of gratifying their revenge. Of this restraint, which madmen have sometimes the power of imposing on their opinions, the remark has been so frequent, that those who are immediately about their persons have termed it in their rude phrase, stifling their disorder." ‡Esquirol‡ makes very similar statements. On the other hand, Dr. A. E. Macdonald § states, that men really insane do not recognize their insanity, and hence do not conceal it. He is, however, the only physician who has had that experience.

Chicago has had recently two striking illustrations of the same kind in the case of Adelaide Roberts, who shot Theo. Weber; she was declared to be insane, was sent to Elgin Hospital, and about two years thereafter released by Judge Rogers under an *habeas corpus* proceeding, and in the case of Mark Gray, the would-be assassin of Edwin Booth, who was declared to be insane, and about two years after was released by Judge Williams, of Quincy, under a similar proceeding.

These learned judges by a stroke of the pen cured these two cases of insanity, after the accomplished superintendent of the Elgin Hospital for the Insane had expended his resources in that direction for two years in vain. Such presumption is marvelous. Had I taken before either of these judges a case of phthisis, and asked him to relieve the patient by the same process, it would have created a doubt, as to my mental soundness, yet insanity is not less a disease than phthisis. The judges would soon recog-

---

* Treatise on Insanity.

† Observation on Madness, p. 53.

‡ Maladies Mentales.

§ *American Journal of Neüroloyy und Psychiatry.* Volume I, p. 120.

nize in a most decided manner the exclusive medical relations of insanity should one of their own family become insane. The judgment of Dr. Kilbourne would then be accepted without question. Neither of them would under such circumstances think for one moment, of calling even upon the most exalted judicial officer for assistence or relief. The case of Adelaide Roberts may on some future occasion be made the basis of some reflections on the medico-legal relations of hysteria and hystero-epilepsy.

At present I ask attention to the case of Mark Gray: May 10, 1879, at an inquest and judgment of the Criminal Court of Cook County, Illinois, Mark Gray was adjudged insane, and committed to the Elgin Hospital for the Insane, into the charge of the superintendent, who was commanded to take the body of the said Mark Gray, and keep it in safety in said asylum until he should have fully and permanently recovered from such insanity. The offense which resulted in this judgment, was an attempt to shoot Edwin Booth, the distinguished tragedian, in McVicker's Theater, Chicago. Mark Gray fired two shots from the dress-circle, and was in the act of firing a third, when he was seized and immediately placed in the custody of the police.

For a day or two after the event he was morose and reticent. He would answer questions, if at all, only in monosyllables. Afterward he became more communicative, and boasted of his wonderful histrionic talent, especially his proficiency in Hamlet, of which he claimed to know every line, and of his ability to render it in a manner much superior to Edwin Booth. His great extravagance in this direction, his excitability, his reticence about the tragedy, the seeming lack of motive for the crime, the fact that Booth had never seen him, led to doubt as to his mental soundness. After a day or two he manifested the delusion which impelled him to the crime. This was the belief that he was the son of Edwin Booth, and as snch, had by heredity his wonderful histrionic talent. Edwin Booth had abandoned him in his childhood, had deprived him of a suitable education for the development of these talents, had neglected his mother, and in revenge for this, Gray shot at him twice, and would have continued firing had he not been arrested. It was at first the opinion of some that there might be a foundation for this belief, notwithstanding its denial by Edwin Booth. Those who entertained this opinion abandoned it when Mrs. Gray made her appearance, a glimpse of her was sufficient to satisfy the most skeptical, more especially as Gray was found to be twenty-eight years old, and it was shown that Mr. Booth had been absent traveling in Australia and elsewhere abroad for two years prior to Gray's birth.

On examination of Gray, three days after the shooting, I found him to be tall and slender, with small muscular development. His face was asymmetrical, the muscles of left side differently inervated from those of the right, so that a smile caused quite a marked distortion of the face. There was twitching of the muscles of the right side of the face. The muscles of the right arm and leg were more active than those of the left. He dragged his left foot in walking and kept the right in more or less activity when talking. His pulse was one hundred and ten and feeble. He complained of headache which had continued for months, and of sleeplessness. His tongue was covered with a heavy white fur and was tremulous.

It was established at the trial in the Criminal Court, that Mark Gray's father died of ascites, four months after Mark's conception, which ascites was probably the result of hepatic cirrhosis, seemingly a consequence of spirit drinking. This is an interesting and important fact in the record. His father was laboring under an incurable disease of nutrition at the time of his conception. The other children of the family have shown none of Mark's peculiarities. It illustates the important bearing of the condition of the parents at the time of the conception upon the health and welfare of the offspring. I recall a case in which the father had been unfortunate in business, left the city, drank heavily, returned home after some days, not yet over his spree, a child was conceived, and it is to-day the inmate of an hospital for the insane, incurably insane. There are four other children in the family, three older and one younger than this patient; all men and women of robust mental and physical health. The spree above mentioned is the only one in which the father ever indulged.

The broken down health of Mark Gray's father at the time of his conception, laid the foundation of a weak nervous organization, which was the first step in the origin of Mark's insanity. Another interesting feature of the case is that Mark's age at the time of the full development of the insanity was about the same as the age of his father, at the time of his death. The disturbance of nutrition, which by attacking abdominal organs gave rise to dropsy in the father, by attacking the brain gave rise to insanity in the son, and this transfer of morbid action from one organ in the parent to another in the progeny is a fact of common observation. Had the same organs been the seat of disease in the son as in the father, the criminal trial never would have taken place, and the stupidity of the Quincy judge would not have manifested itself in this direction.

Mark Gray grew up possessed of inordinate conceit and exalted self feeling and having ideas of grandeur and importance. In early life he became intemperate, a part of the time he drank heavily. In 1876 he stopped drinking excessively, and began to act strangely about his home. He would get up at night and declaim Shakespeare the night through, He would keep himself away from the other members of the family, and would sit for hours with his head between his hands. At other times he would strike "stage attitudes" and remain for a long time in these. So peculiar was his conduct, that his mother and sister were much alarmed about him. It was at this time that he conceived the delusion of his relationship to Edwin Booth. He told me that he heard it frequently whispered as he passed along the street, "there goes the bastard son of Booth." His fellow-clerks in the store tormented him by the same sort of whispering. These were evidently auditory hallucinations.

After a time he determined to have an interview with Mr. Booth, and demand a monetary compensation for the years of imaginary neglect which he had sustained. For this purpose he came to Chicago, April 22, 1879, and went immediately to the theatre to see Mr. Booth, but did not find him there. He went to the theatre again that night. Mr. Booth was playing Richelieu. During this play Gray imagined that Mr. Booth saw him sitting in the gallery and recognized him; made faces at him, called him by name several times, "Mark! Mark!" and made fun of his mother. Gray left the theatre with the resolution to kill Booth, for these insults, the next night. He purchased a pistol the following day, and afterwards secured the seat which he thought would serve his purpose best, in the dress-circle near the stage, and by a study of the play, selected the prison scene as a favorable time for firing the shots. He was arrested, tried, and found to be insane as already stated.

At the Elgin Hospital for the Insane, his delusion of his relationship to Booth, his delusion of his wonderful histrionic talent, and his constant reading and declaiming Shakespeare were manifest. December 15, 1879, he importuned Dr. Kilbourne for his discharge, as he had often done before. Dr. Kilbourne told him that he was still insane and, as a proof of it, stated that his (Gray's) delusion of being Edwin Booth's son was just as fixed as the day he entered the hospital. The day after this the hospital record shows that Gray gave up reading and declaiming Shakespeare,and when spoken to about being the son of Booth, would say that he had given up all idea of such a relationship, that it was a crazy notion of which he had rid himself. He continued then to assert on all occasions, that his delusions had been corrected, and he manifested no interest in Shakespeare or theatrical matters, until December 1, 1880.

During this interval of nearly one year, the Board of Trustees had Mark before them three or four times, carefully examined into his mental state, and thought he might be discharged, but Dr. Kilbourne, not being quite satisfied, asked them to wait a short time longer. December 1, 1880, Gray broke out afresh, and the hospital notes show "that he refuses to have his hair cut, likes to wear it long, as it looks more stage-like, practises elocution every day in his room, considers himself a great Shakesperian scholar, has talent for the stage much superior to that of his father Booth." He continued thus to manifest his delusions every day. Thus, on October 18, 1881, in conversation with Dr. Crane, assistant physician of the Hospital, Gray said that Mark Lyon (Gray's father) was Edwin Booth ; that his father's brother, Pat Lyon, was Junius Brutus Booth, Jr. ; another brother of his father was John Wilkes Booth, and still another, Bryan Lyon, was Joseph Murphy the comedian, whom he believes is a brother of the Booth's. He does not believe that Wilkes Booth is dead. He believes his cousin Mary Lyon, is a daughter of Junius Brutus Booth.

February 5, 1882, he had an interview with the Board of Trustees seeking a release from the hospital. In that interview he was again told that so long as he had the delusions concerning Booth and the stage, he could not be discharged. During this interview he was much excited, violent at times, and incoherent in language. He left the room evidently resolved on concealment again, for he ceased from that time to manifest any interest in theatrical matters, and laughed at his delusion concerning Booth, so that his attendant, who was employed shortly after this event, and who was quite constantly with him for about eight months, saw at no time any evidence of mental disturbances, but noticed Gray avoided with much effort and with a surprising degree of indifference any reference to theatrical matters.

The *habeas corpus* trial occurred in Quincy. Why in Quincy, two hundred miles from the place of the criminal trial, is a mystery! The trial was conducted as such trials usually are. A dozen people were called by Mark's attorney. Some had known him before; then talked to him for a few minutes about the weather, business, politics, theatres, etc., and all with one accord testified, that they had found no evidence of insanity about him. This purely negative evidence would release from custody nine-tenths of the patients of any hospital for the insane. Four persons who were announced as physicians were called by Gray's attorney. One, a veterinary surgeon, who felt quite confident of Gray's complete restoration. One, a retired clergyman, who had attended one course of lectures in a medical school, who, to his credit be it said, testified that if the patient had deceived the hospital authorities as to his insanity for one year, it is quite probable that he might be doing it now, and he would hesitate therefore in aiding Gray's discharge. One was a young M.D., son of the retired clergyman, before mentioned, fresh from a medical college, who will probably be wiser when he is older, and the fourth was a physician of fine attainments, with that familiarity with insanity which the country practitioner has. He testified that while he saw no evidence of insanity in Gray, yet he would not advise his discharge against the judgment of Dr. Kilbourne. In addition, an attendant who came to the hospital about one month after Mark began to conceal his delusions, the last time, testified, that he had daily intercourse with him and had observed no evidence of insanity, but was surprised at the pertinacity with which he avoided all conversation upon theatrical matters, the attendant being much interested in such things.

Mark testified in his own behalf, and his testimony was a surprise to the newspaper reporters and the people about the court room. The average individual looks upon insanity as a complete loss of reasoning powers, as something which must be violent and striking in its demonstrations. I have repeatedly taken visitors through the Insane Hospital of which I was the superintendent, and when every ward had been visited have them ask me to show them the lunatics. To one familiar with insanity, and the history of this case, Mark's own testimony was sufficient to show that the disease was not eradicated. He indulged in that denunciation of the Hospital authorities, which is usual in such cases. He pronounced judgment on the assistant physician, Dr. Crane, now in private practice in New York, to the effect that *he* was crazy, "crazier than witness was." Dr. Kilbourne, one of the most successful superintendents in the West, a thoroughly scientific physician, was to him vile and despicable. He accused Dr. Kilbourne of taunting him with the story of his birth ; of abusing him so maliciously and acting in his visits to him so like a crazy man, that after he went out the attendant told him he ought to have knocked Dr. Kilbourne down. I have often, as has every superintendent of an insane hospital, heard precisely such abuse from this class of patients. It is a remarkable fact, that patients who leave the hospital cured, always have pleasant recollections of those who cared for them in their affliction. This unwarranted abuse of Drs. Kilbourne and Crane would be sufficient to establish Gray's insanity were there no other evidences of it. Gray told the story of his life and of the great tragedy he tried to enact, with a smile on his lips and with many efforts at jocularity; when these jokes provoked laughter in the hangers-on of the court, Gray seemed particularly happy. He told with evident delight of the way he had fooled all the asylum authorities for one year; that Dr. Kilbourne had told him he could not be discharged until he had given up his delusions; that he then resolved to conceal them; that he was successful in deceiving his ward attendant, Dr. Crane, and the Board of Trustees, and after playing the game, as he expressed it, for a year, he gave it up. The learned judge, at this part of Mark's testimony, asked him: "If you admit that you did practice this deception for one year, how shall I know that you are not doing it now?" Mark, after a long hesitation, answered: "I don't know," and in a very tragic attitude, rising from his chair, appealed to God to witness that he was not fooling now. On behalf of the hospital, Mr. J. S. Miller, the attorney, first presented the record of Mark's hospital life; an abstract taken from the daily reports of his various attendants, showing the presence of his delusions; the concealment of them for one year; the subsequent reappearance of them in the same form, and the concealment beginning in February, 1882. Dr. Kilbourne testified to the same effect, and stated in strong and positive language his belief that Mark Gray was still insane. Dr. W. A. Byrd, one of the leading surgeons of Quincy, after hearing all the testimony, and after a careful personal study of Gray, testified that he was then insane. Dr. Byrd dwelt upon the evidence of neurosis, as shown in asymmetry of the face, in unequal action of the muscles of the two sides, in the twitching of the facial muscles, and those of the shoulder and hand.

My testimony, and that of Mr. Rice, a deputy sheriff of Cook Co., who had taken Mark to the hospital, and had frequently seen him there and knew of his concealed delusion, was to the same effect. Judge Williams then rendered his opinion, releasing Gray from restraint.

Of course there could be no doubt that the relator was insane, if there had been any doubt, his subsequent history placed it beyond question. He wrote a letter four weeks ago to Mr. John W. Norton, of the Grand Opera House, St. Louis, in which he proposed to star Hamlet in small towns; still later to the Keokuk correspondent of the Chicago *Tribune* he said: "It is my intention to make arrangements to star with a company on the road. The notoriety I have achieved during the last few years and my great resemblance to Booth would draw crowded houses. I resemble Mr. Booth in every particular except the eyes; his are deeply sympathetic, mine the most brilliant. Of course my going on the stage will hurt Booth," and much more talk of the same character. The resemblance to Mr. Booth is an insane fancy.

This case suggests the necessity of taking this matter of discharging criminal lunatics out of the power of the judiciary. It should be placed in the hands of those who have given some personal attention and study to insanity; those who recognize the fact that insanity can be concealed, and the further important fact that the homicidal impulse may lie dormant for years, and then manifest itself in its former fury. Hadfield, whose case is quoted in every book on medical jurisprudence, who was released by the eloquence of Erskine, from the responsibility for his act of firing at George III., in Drury Lane Theatre, remained in the Bethlehem Hospital for the Insane for years before he showed any other homicidal impulse, and then he made a murderous assault upon a keeper for whom he had always manifested the greatest regard. In Illinois, this important matter could with safety be confided to the State Board of Public Charities.

[NOTE.—I am very much indebted to Dr. Jas. G. Kiernan for citations of authorities found in this paper.]—*Alienist and Neurologist.*

